# Care complexity individual factors associated with adverse events and in-hospital mortality

**DOI:** 10.1371/journal.pone.0236370

**Published:** 2020-07-23

**Authors:** Jordi Adamuz, Maria-Eulàlia Juvé-Udina, Maribel González-Samartino, Emilio Jiménez-Martínez, Marta Tapia-Pérez, María-Magdalena López-Jiménez, Marta Romero-Garcia, Pilar Delgado-Hito

**Affiliations:** 1 Nursing knowledge management and information systems department, Bellvitge University Hospital, Bellvitge Institute of Biomedical Research (IDIBELL), L’Hospitalet de Llobregat, Barcelona, Spain; 2 School of Nursing, Medicine and Health Science Faculty, University of Barcelona, Bellvitge Institute of Biomedical Research (IDIBELL), L’Hospitalet de Llobregat, Barcelona, Spain; 3 Catalan Institute of Health, Barcelona, Spain; 4 Infectious Disease Department, Bellvitge University Hospital, Bellvitge Institute of Biomedical Research (IDIBELL), L’Hospitalet de Llobregat, Barcelona, Spain; University of Oxford, UNITED KINGDOM

## Abstract

**Introduction:**

Measuring the impact of care complexity on health outcomes, based on psychosocial, biological and environmental circumstances, is important in order to detect predictors of early deterioration of inpatients. We aimed to identify care complexity individual factors associated with selected adverse events and in-hospital mortality.

**Methods:**

A multicenter, case-control study was carried out at eight public hospitals in Catalonia, Spain, from January 1, 2016 to December 31, 2017. All adult patients admitted to a ward or a step-down unit were evaluated. Patients were divided into the following groups based on the presence or absence of three adverse events (pressure ulcers, falls or aspiration pneumonia) and in-hospital mortality. The 28 care complexity individual factors were classified in five domains (developmental, mental-cognitive, psycho-emotional, sociocultural and comorbidity/complications). Adverse events and complexity factors were retrospectively reviewed by consulting patients’ electronic health records. Multivariate logistic analysis was performed to identify factors associated with an adverse event and in-hospital mortality.

**Results:**

A total of 183,677 adult admissions were studied. Of these, 3,973 (2.2%) patients experienced an adverse event during hospitalization (1,673 [0.9%] pressure ulcers; 1,217 [0.7%] falls and 1,236 [0.7%] aspiration pneumonia). In-hospital mortality was recorded in 3,996 patients (2.2%). After adjustment for potential confounders, the risk factors independently associated with both adverse events and in-hospital mortality were: mental status impairments, impaired adaptation, lack of caregiver support, old age, major chronic disease, hemodynamic instability, communication disorders, urinary or fecal incontinence, vascular fragility, extreme weight, uncontrolled pain, male sex, length of stay and admission to a medical ward. High-tech hospital admission was associated with an increased risk of adverse events and a reduced risk of in-hospital mortality. The area under the ROC curve for both outcomes was > 0.75 (95% IC: 0.78–0.83).

**Conclusions:**

Several care complexity individual factors were associated with adverse events and in-hospital mortality. Prior identification of complexity factors may have an important effect on the early detection of acute deterioration and on the prevention of poor outcomes.

## Introduction

The World Health Organization reports that thousands of people are affected by complications and adverse events (AEs) associated with caregiving, and that these events increase morbidity and mortality rates worldwide [1–3]. Complications in hospital are unfortunate for patients and expensive for healthcare systems [4]. Health organizations stress the importance of establishing a culture of safety to ensure that patients are not inadvertently harmed by care errors. AEs are incidents in which harm is caused to a person receiving health care, generally resulting in additional treatment, prolonged hospital stay, disability at the time of discharge, or death [5]; they have become a global problem and an important indicator of patient safety [6].

Studies including large patient samples estimate that approximately 10% of hospital admissions are associated with an AE [7,8] and that 4% of deceased inpatients experience preventable AEs prior to death [9,10]. Pressure ulcers, falls, aspiration pneumonia and other AEs are associated with causative factors such as age, clinical complexity, co-morbidity, illness severity, reduced functional activity and lower quality of care [11]. In this regard, nurses play a key role in implementing strategies for preventing functional and cognitive decline [4,12]. A range of preventive nursing interventions are recommended to treat the three AEs mentioned: skin care to avoid pressure ulcers, patient orientation to avoid falls, and swallowing assessment to avoid aspiration pneumonia. Failure to apply preventive interventions may lead to functional and cognitive decline, which are considered preventable risk factors for the development of cascade iatrogenesis [4,13].

Patients in tertiary care hospitals today present higher complexity than in the past, and the degree of complexity varies greatly from case to case [14]. Complex patients are more vulnerable to complications and are often burdened by multiple chronic conditions and psychological issues [15]. The term “complexity of care” has been widely used in international healthcare, and has been applied to patients with functional and health limitations and also social or non-medical issues [16]. Therefore, patients may be complex not only due to multiple co-occurring medical conditions, but also due to behavioral and psychosocial factors that often represent a major barrier to achieving optimal health. In this context, the Vector Model of Complexity defines the determinants of complexity, along axes representing major health determinants [17]. Juvé-Udina et al. also identified care complexity individual factors (CCIF) in hospitalized patients, classifying them into five domains: developmental, mental-cognitive, psycho-emotional, sociocultural and comorbidity/complications. In their qualitative participatory action study involving 404 nurses of eight public hospitals, the results identified four domains that coincided with the Vector Model of Complexity [18].

Similarly, the measurement of the impact of care complexity on healthcare outcomes is a key issue that requires comprehensive evaluation. Some studies have assessed specific care models in order to predict increases in medical needs during hospitalization, emergency department visits, or 30-day readmission rates [17,19]. These studies concluded that more work focusing on psychosocial, biological and environmental aspects is needed to identify patients at risk of AEs or mortality [17]. Determining these non-medical factors, which include psychosocial components such as self-management and self-care abilities, may help in the design of multicomponent interventions that can then be tested in hospitalized patients [16]. There is a growing interest in the relationship between CCIF and healthcare outcomes such as AEs or and in-hospital mortality, and the aim of the present study was to explore this association in depth.

## Material and methods

### Setting and study design

A case-control study was carried out at eight public hospitals affiliated to the Catalan Institute of Health, the major public healthcare provider in Catalonia, Spain: three high-tech metropolitan facilities, three urban university centers and two community hospitals. The hospitals serve an area of 5,500,000 inhabitants and discharge around 150,000 patients per year. All patients older than 17 years admitted to a ward or step-down unit from January 1, 2016 to December 31, 2017, and with a completed nursing assessment, were retrospectively recruited after discharge and follow-up using the information charted in the electronic health records. Patients admitted to palliative care, critical care and obstetrics units were excluded.

For this study, patients were classified into the following groups: those who had AEs (pressure ulcers, falls or aspiration pneumonia not present on admission) during hospitalization (presence of AEs); those who did not (absence of AEs); and those who died in hospital (in-hospital mortality).

This study was evaluated and approved by the Clinical Research Ethics Committee (CEIC) of the Bellvitge University Hospital (reference 114/17). Informed consent was waived due to the study’s retrospective design. Ethical and data protection protocols related to anonymity and data confidentiality (access to records, data encryption and archiving of information) were observed throughout the whole research process.

### Data collection

Information regarding the demographic characteristics, unit of admission (medical or surgical ward), continuity of care (discharged/not discharged to another facility), high-tech hospital (referral center that provides tertiary care for either open-heart surgery or major organ transplants or both, or other center), length of hospital stay and relative weight of diagnosis-related group (DRG) were collected from the electronic health record system, the hospital minimum data set and the clinical data warehouse of the Catalan Institute of Health (*Institut Català de la Salut*).

Medical wards were defined as those admitting patients for health conditions that required medical diagnostic or therapeutic interventions and patients who would require short-term continuity of care at home. Surgical wards included those that admitted patients for health conditions requiring any surgical procedure, including all surgical specialties and organ transplants. Step-down units were pre- and/or post-intensive care wards, offering highly specialized treatment and close monitoring in order to fulfill an intermediate role between the standard care unit and the intensive care unit (ICU).

The diagnosis-related group (DRG) included estimators of resource consumption and costs, known as relative weights [20]. The DRG system attributes a relative weight to each DRG based on its cost, with the value of 1 representing the mean global cost. In Spain, these weights and costs are derived from information on hospital care costs obtained by the hospital accounting systems [21,22]. Moreover, the DRG system assigns a severity and mortality risk according to all patient refined diagnosis-related groups (APR-DRG) used to label patients in the minimum data set from low (level 1) to extreme (level 4). Severity and mortality risk were dichotomized in this study into low risk (1–2) and high risk (3–4). All the variables collected were selected according to the medical and nursing scientific literature [18,19,23].

For this study we examined the following three AEs not present on admission which occur frequently in hospitals and are considered to be sensitive to nursing care [4,6]: pressure ulcers (at any stage), falls (with or without visible injuries) and aspiration pneumonia (pneumonitis due to inhalation of food or vomit).

Care complexity individual factors are a set of characteristics related to different health dimensions that may complicate care delivery [18] and may contribute to adverse outcomes. CCIF are classified into five domains: (i) mental-cognitive, (ii) psycho-emotional, (iii) sociocultural, (iv) developmental, and (v) comorbidity/complications. Each CCIF domain is structured into factors and specifications. Nurses assessed patients at admission and on an ongoing basis in order to monitor changes in clinical status. Non-modifiable CCIF (i.e., extreme age) were collected solely at admission, while modifiable CCFI (i.e., hemodynamic instability) were collected for every patient day from admission to discharge. The specifications of CCIF were part of the coded and structured data in the initial and ongoing nursing assessment sections of the electronic health record, as described in the ATIC (Architecture, Terminology, Interface, Information, Nursing and Knowledge) terminology [24]. Patients were considered to present CCIF domains if they presented at least one related factor or specification. All data were obtained from patients' electronic health records and were collected blindly.

The mental-cognitive domain included four factors: (i) agitation, (ii) mental status impairments (confusion, disorientation, stupor, transient loss of consciousness), (iii) impaired cognitive functions (intellectual disability, amnesia) and (iv) perception of reality disorders (delirium, hallucinations, disconnection from reality). The psycho-emotional domain comprised three factors: (i) aggressive behavior, (ii) fear/anxiety and (iii) impaired adaptation (disruptive behavior, hopelessness or surrender). The sociocultural domain included four factors: (i) language barriers, (ii) social exclusion (extreme poverty), (iii) belief conflict (spiritual distress), (iv) lack of caregiver support. The developmental domain encompassed two factors: (i) old age (≥75 years old) and (ii) adolescence (17–19 years old). Finally, the comorbidity/complications domain contained 15 factors: (i) major chronic disease, (ii) hemodynamic instability (intensive control of vital signs or state of shock), (iii) high risk of hemorrhage (coagulation disorders, thrombocytopenia, anticoagulant therapy), (iv) communication disorders (aphasia, dysphasia, dysarthria, laryngectomy, tracheostomy), (v) urinary or fecal incontinence, (vi) vascular fragility (capillary fragility, tortuous veins), (vii) position impairment, (viii) involuntary movements (continuous involuntary movements), (ix) extreme weight (low weight, obesity), (x) dehydration (skin turgor), (xi) edema, (xii) uncontrolled pain (verbal numerical rating scale above three points), (xiii) transmissible infections (isolation measures), (xiv) immunosuppression and (xv) anatomical and functional disorders (amputation, deformities, joint stiffness).

### Data analyses

Descriptive analysis of data using percentage frequencies, median and interquartile range was performed to describe patients’ demographic and clinical characteristics and their outcomes. For categorical variables, a comparative analysis for detecting significant differences between groups was carried out using the chi-square test or Fisher’s exact test when one or more cells had an expected frequency of five or less. For continuous variables, the Student’s t-test or Mann-Whitney U test was used depending on the results of the Kolmogorov-Smirnov normality test. The logistic-regression model of factors potentially associated with AEs and in-hospital mortality included the 28 CCIF and possible confounders (gender, length of stay, high-tech hospital, and medical ward). A logistic-regression model of each AE was also performed. All the 28 CCIF and potential confounders included in the multivariate analyses were subjected to a correlation matrix for analysis of collinearity. The number of variables included in the multivariate analysis was restricted following the rule that there should be at least ten events per variable [25]. The discriminatory power was evaluated by the area under the receiver operating characteristic (ROC AUC) and its 95% confidence interval (CI). An AUC of 0.5 indicates that the model has a predictive discrimination no better than chance, whereas an AUC of 1.0 indicates a perfectly discriminating model. Commonly, an AUC of 0.5–0.7 is interpreted as a model with low discriminatory power, 0.7–0.9 moderate and > 0.9 high discriminatory power. The results of the multivariate analyses were reported as odds ratios (OR) and 95% confidence intervals (CI). Finally, the individual risk of each patient’s outcome according to the number of CCIF was assessed by means of a chi-square analysis for linear trends. P values less than 0.05 were considered statistically significant. All reported p values are two-tailed. Statistical analysis was performed using the SPSS software package version 25.0 (SPSS, Chicago, IL).

## Results

During the study period, 183,677 adult patients were admitted to a hospital ward or step-down unit. Adverse events recorded in 3,973 (2.2%) patients during hospitalization (1,673 [0.9%] pressure ulcers; 1,217 [0.7%] falls and 1,236 [0.7%] episodes of aspiration pneumonia). The frequency of in-hospital mortality was 2.2% (3,996 patients). Half of the patients were male, admitted to medical wards, in high-tech hospitals and with unscheduled admission. Just over 7% were admitted to step-down units and nearly 6% to ICUs. The median hospital stay was four days, and 26% of patients had a high risk of severity or mortality (APR-GRD 3–4) (Table 1).

**Table 1:** Baseline characteristics of adult patients according to adverse events and in-hospital mortality.

Characteristic	Study population n = 183,677		No adverse event n = 179,704 (97.8)		Adverse event n = 3,973 (2.2)		Pressure ulcer n = 1,673 (0.9)		Falls n = 1,217(0.7)		Aspiration pneumonia n = 1,236 (0.7)		In-hospital mortality n = 3,996 (2.2)	
	No.	(%)	No.	(%)	No.	(%)	No.	(%)	No.	(%)	No.	(%)	No.	(%)
Age (years)_median (IQR)	67	(53–78)	66.9	(52–78)	76	(66–84)	78	(68–85)	74	(64–82)	77	(62–85)	80.7	(71–87)
Age ≥ 75 years	58,005	(31.6)	55,823	(31.1)	2,182	(54.9)	994	(59.4)	623	(51.2)	682	(55.2)	2,652	(66.4)
Male sex	102,764	(55.9)	100,426	(55.9)	2,338	(58.8)	943	(56.4)	752	(61.8)	744	(60.2)	2,306	(57.7)
Medical ward	96,058	(52.3)	93,301	(51.9)	2,757	(69.4)	1,092	(65.3)	815	(67.0)	955	(77.3)	3,222	(80.6)
Psychiatric ward	608	(0.3)	584	(0.3)	24	(0.6)	6	(0.4)	13	(1.1)	6	(0.5)	2	(0.1)
Surgical ward	87,619	(47.7)	86,403	(48.1)	1,216	(30.6)	581	(34.7)	402	(33.0)	281	(22.7)	774	(19.4)
Step-down unit	13.582	(7.4)	12,894	(7.2)	688	(17.3)	296	(17.7)	149	(12.2)	268	(21.7)	464	(11.6)
ICU admission	10,894	(5.9)	10,142	(5.6)	752	(18.9)	311	(18.6)	160	(13.1)	326	(26.4)	680	(17.0)
Unscheduled admission	101,749	(55.4)	98,413	(54.8)	3,336	(84)	1,373	(82.1)	938	(77.1)	1,160	(93.9)	3,603	(90.2)
Length of stay_median (IQR)	4	(2–8)	4	(2–8)	14	(8–25)	17	(10–32)	14	(8–23)	11	(6–19)	6	(3–13)
High-tech hospital	104,264	(56.8)	101,663	(56.6)	2,601	(65.5)	1,060	(63.4)	810	(66.6)	828	(67.0)	2,109	(52.8)
Continuity of care^a^	7.330	(4.0)	6,755	(3.8)	575	(14.5)	297	(17.8)	102	(8.4)	202	(16.3)	-	-
Relative median weigh of DRG_average (SD)	1.2	(1.2)	1.2	(1.1)	2.3	(2.7)	2.4	(3.0)	1.8	(2.1)	2.8	(2.7)	2.2	(2.4)
Severity or mortality (APR-GRD 3–4)	48,069	(26.2)	45,089	(25.1)	2,980	(75)	1,250	(74.7)	662	(54.4)	1,212	(98.1)	3,510	(87.8)

Abbreviations: IQR, interquartile range; SD, standard deviation; APR-DRG, all patient refined diagnosis-related groups.

^a^ Continuity of care was defined as discharged to another facility.

### Patient characteristics

The demographic and clinical characteristics of patients with and without AE and in-hospital mortality are compared in Table 1. Patients with an AE during hospitalization were more often male, older (≥75 years) and more likely to be admitted to a medical ward. They were also more frequently admitted to a high-tech hospital, needed care in step-down units or ICUs, required unscheduled admission and presented longer hospital stay. Likewise, patients who had an AE more often had a higher risk of severity or mortality during hospitalization and needed more continuity of care after discharge. Similar results were found for patients who died during hospitalization. Although patients admitted in high-tech hospitals and surgical wards presented less frequency of in-hospital mortality, compared with patients who had an AE.

### CCIF according to AEs and in-hospital mortality

CCIF in admitted patients with the three selected AEs (pressure ulcers, falls and aspiration pneumonia) and in-hospital mortality are compared in Table 2. Patients with an AE were more likely to have CCIF in the comorbidity/complications, developmental, mental-cognitive, psycho-emotional, and sociocultural domains. Regarding comorbidity/complications, hemodynamic instability, major chronic disease and uncontrolled pain were the most frequent CCIF identified in patients with AEs. In the other domains, the most frequently factors were extreme age (≥75 years old) in the developmental domain, impaired adaptation in the psycho-emotional domain, mental status impairments in the mental-cognitive domain and lack of caregiver support in the sociocultural domain. The only complexity factors that were not associated with AEs were dehydration and language barriers. Similar results were found for all three AEs.

**Table 2:** Care complexity individual factors of adult hospitalized patients according to adverse events and in-hospital mortality.

Care complexity individual domains/factors	Study population		No adverse event		Adverse event		Pressure ulcer		Falls		Aspiration pneumonia		In-hospital mortality	
	n = 183,677		n = 179,704 (97.8)		n = 3,973 (2.2)		n = 1,673 (0.9)		n = 1,217(0.7)		n = 1,236 (0.7)		n = 3,996 (2.2)	
	N	(%)	N	(%)	N	(%)	N	(%)	N	(%)	N	(%)	N	(%)
**Mental-cognitive**	**12,126**	**(6.6)**	**1,0843**	**(6.0)**^**a**^	**1,283**	**(32.3)**^**a**^	**527**	**(31.5)**^**a**^	**250**	**(20.5)**^**a**^	**575**	**(46.5)**^**a**^	**1,488**	**(37.2)**^**a**^
Agitation	1,628	(0.9)	1,469	(0.8)^**a**^	159	(4.0)^**a**^	55	(3.3)^**a**^	44	(3.6)^**a**^	73	(5.9)^**a**^	143	(3.6)^**a**^
Mental status impairments	10,924	(5.9)	9,719	(5.4)^**a**^	1,205	(30.3)^**a**^	503	(30.1)^**a**^	229	(18.8)^**a**^	537	(43.4)^**a**^	1,425	(35.7)^**a**^
Impaired cognitive functions	437	(0.2)	413	(0.2)^**a**^	24	(0.6)^**a**^	8	(0.5)	2	(0.2)	14	(1.1)^**a**^	8	(0.2)
Perception of reality disorders	437	(0.2)	401	(0.2)^**a**^	36	(0.9)^**a**^	25	(1.5)^**a**^	6	(0.5)^**a**^	7	(0.6)^**b**^	30	(0.8)^**a**^
**Psycho-emotional**	**21,347**	**(11.6)**	**20,276**	**(11.3)**^**a**^	**1,071**	**(27.0)**^**a**^	**417**	**(24.9)**^**a**^	**287**	**(23.6)**^**a**^	**418**	**(33.8)**^**a**^	**948**	**(23.7)**^**a**^
Aggressive behavior	553	(0.3)	511	(0.3)^**a**^	42	(1.1)^**a**^	19	(1.1)^**a**^	8	(0.7)^**b**^	17	(1.4)^**a**^	17	(0.4)
Fear/anxiety	14.650	(8)	14,094	(7.8)^**a**^	556	(14)^**a**^	206	(12.3)^**a**^	174	(14.3)^**a**^	199	(16.1)^**a**^	430	(10.8)^**a**^
Impaired adaptation	8,436	(4.6)	7,795	(4.3)^**a**^	641	(16.1)^**a**^	253	(15.1)^**a**^	151	(12.4)^**a**^	270	(21.8)^**a**^	600	(15)^**a**^
**Sociocultural**	**6,308**	**(3.4)**	**5,853**	**(3.3)**^**a**^	**455**	**(11.5)**^**a**^	**189**	**(11.3)**^**a**^	**125**	**(10.3)**^**a**^	**167**	**(13.5)**^**a**^	**433**	**(10.8)**^**a**^
Language barriers	759	(0.4)	748	(0.4)	11	(0.3)	1	(0.1)^**b**^	8	(0.7)	3	(0.2)	9	(0.2)
Social exclusion	145	(0.1)	135	(0.1)^**a**^	10	(0.3)^**a**^	2	(0.1)^**b**^	4	(0.3)^**b**^	5	(0.4)^**b**^	5	(0.1)
Belief conflict	173	(0.1)	162	(0.1)^**a**^	11	(0.3)^**a**^	5	(0.3)	4	(0.3)	2	(0.2)	10	(0.3)^**b**^
Lack of caregiver support	5,461	(3)	5,022	(2.8)^**a**^	439	(11) ^**a**^	184	(11.0)^**a**^	118	(9.7)^**a**^	163	(11.0)^**a**^	418	(10.5)^**a**^
**Developmental**	**59,069**	**(32.2)**	**56,879**	**(31.7)**^**a**^	**2,190**	**(55.1)**^**a**^	**997**	**(59.6)**^**a**^	**597**	**(49.1)**^**a**^	**685**	**(55.4)**^**a**^	**2,653**	**(66.4)**^**a**^
Old age (≥75 years)	58,005	(31.6)	55,823	(31.1)^**a**^	2,182	(54.9)^**a**^	994	(59.4)^**a**^	594	(48.8)^**a**^	682	(55.2)^**a**^	2,652	(66.4)^**a**^
Adolescence (17–19 years)	1,064	(0.6)	1,056	(0.6) ^**a**^	8	(0.2)^**a**^	3	(0.2)^**b**^	3	(0.2)	3	(0.2)	1	(0.0)^**a**^
**Comorbidity/complications**	**155,547**	**(84.7)**	**151,717**	**(84.4)**^**a**^	**3,830**	**(96.4)**^**a**^	**1,636**	**(97.8)**^**a**^	**1,167**	**(95.9)**^**a**^	**1,178**	**(95.3)**^**a**^	**3,902**	**(97.6)**^**a**^
Major chronic disease	76,869	(41.9)	74,788	(41.6)^**a**^	2,081	(52.4)^**a**^	911	(54.5)^**a**^	660	(54.2)^**a**^	589	(47.7)^**a**^	1,772	(69.4)^**a**^
Hemodynamic instability	110,309	(60.1)	107,094	(59.6)^**a**^	3,215	(80.9)^**a**^	1,370	(81.9)^**a**^	1,003	(82.4)^**a**^	977	(79.0)^**a**^	2,910	(72.8)^**a**^
High-risk of hemorrhage	4,233	(2.3)	4,117	(2.3)^**b**^	116	(2.9)^**b**^	44	(2.6)	45	(3.7)^**b**^	32	(2.6)	122	(3.1)^**b**^
Communication disorders	7,524	(4.1)	6,737	(3.7)^**a**^	787	(19.8)^**a**^	283	(16.9)^**a**^	133	(10.9)^**a**^	411	(33.3)^**a**^	720	(18.0)^**a**^
Urinary or fecal incontinence	14,381	(7.8)	13,326	(7.4)^**a**^	1,055	(26.6)^**a**^	519	(31.0)^**a**^	200	(16.4)^**a**^	384	(31.1)^**a**^	1,114	(27.9)^**a**^
Vascular fragility	3,937	(2.1)	3,670	(2.0)^**a**^	267	(6.7)^**a**^	139	(8.3)^**a**^	60	(4.9)^**a**^	79	(6.4)^**a**^	396	(9.9)^**a**^
Position impairment	5,291	(2.9)	4,932	(2.7)^**a**^	359	(9.0)^**a**^	196	(11.7)^**a**^	77	(6.3)^**a**^	111	(9.0)^**a**^	293	(7.3)^**a**^
Involuntary movements	842	(0.5)	780	(0.4)^**a**^	62	(1.6)^**a**^	24	(1.4)^**a**^	14	(1.2)^**b**^	30	(2.4)^**a**^	43	(1.1)^**a**^
Extreme weight	8,322	(4.5)	7,990	(4.4)^**a**^	332	(8.4)^**a**^	190	(11.4)^**a**^	59	(4.8)	100	(8.1)^**a**^	339	(8.5)^**a**^
Dehydration	99	(0.1)	96	(0.1)	3	(0.1)	1	(0.1)	1	(0.1)	1	(0.1)	17	(0.4)^**a**^
Edema	1,775	(1)	1,686	(0.9)^**a**^	89	(2.2)^**a**^	52	(3.1)^**a**^	22	(1.8)^**b**^	20	(1.6)^**b**^	154	(3.9)^**a**^
Uncontrolled pain	49,332	(26.9)	47,742	(26.6)^**a**^	1,590	(40)^**a**^	755	(45.1)^**a**^	539	(44.3)^**a**^	367	(29.7)^**b**^	1,388	(34.7)^**a**^
Transmissible infection	6,024	(3.3)	5,526	(3.1)^**a**^	498	(12.5)^**a**^	317	(18.9)^**a**^	115	(9.4)^**a**^	96	(7.8)^**a**^	276	(6.9)^**a**^
Immunosuppression	889	(0.5)	860	(0.5)^**b**^	29	(0.7)^**b**^	5	(0.3)	15	(1.2)^**b**^	9	(0.7)	23	(0.6)
Anatomical and functional disorders	18,663	(10.2)	17,963	(10.0)^**a**^	700	(17.6)^**a**^	360	(21.5)^**a**^	189	(15.5)^**a**^	179	(14.5)^**a**^	557	(13.9)^**a**^
CCIF, median (IQR)	2	(1–3)	2	(1–3)^**a**^	4	(3–5)^**a**^	4	(3–6)^**a**^	3	(2–5)^**a**^	4	(3–6)^**a**^	4	(3–5)^**a**^
CCIF = 1	45,960	(25)	45,709	(25.4)^**a**^	251	(6.3)^**a**^	81	(4.8)^**a**^	87	(7.1)^**a**^	87	(7.0)^**a**^	168	(4.2)^**a**^
CCIF = 2 or 3	83,869	(45.7)	82,463	(45.9)^**a**^	1,406	(35.4)^**a**^	540	(32.3)^**a**^	545	(44.8)	364	(29.4)^**a**^	1,376	(34.4)^**a**^
CCIF ≥ 4	33,140	(18)	30,890	(17.2)^**a**^	2,250	(56.6)^**a**^	1,035	(61.9)^**a**^	561	(46.1)^**a**^	760	(61.5)^**a**^	2,432	(60.9)^**a**^

Abbreviations: IQR, interquartile range. CCIF, care complexity individual factors.

^a^ p value ≤0.001.

^b^ p value >0.001 and <0.05.

Moreover, several factors from the comorbidity/complication, developmental and mental-cognitive domains were more frequent in patients who died during hospitalization than in patients who experienced an AE. These factors were major chronic disease, old age, mental status impairments, urinary or fecal incontinence, vascular fragility, extreme weight, edema, high-risk of hemorrhage and dehydration.

**Fig 1:**
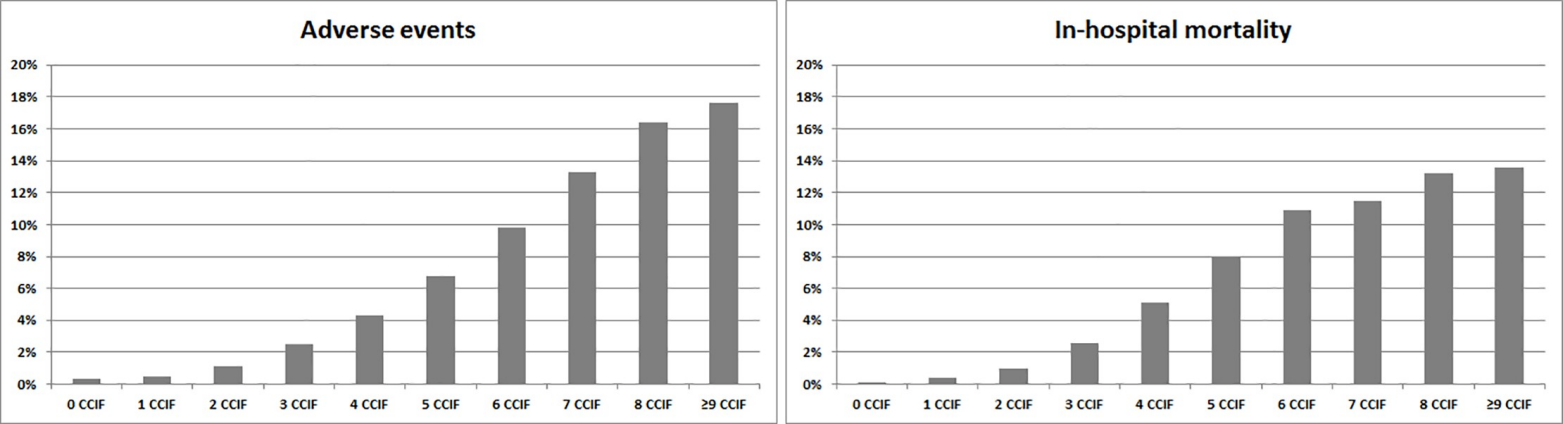
Proportions of AE and in-hospital mortality according to the number of care complexity individual factors.

Of the 183,677 patients analyzed in this study, 117,009 (63.7%) had two or more individual complexity factors. AEs and in-hospital mortality were more frequently identified in patients with at least four CCIF. Four or more CCIF were identified in 2,250 patients with an AE (56.6%) and in 2,432 patients who died during hospitalization (60.9%). The frequency of AEs rose with the increasing number of CCIF and reached almost 10% in patients with six complexity factors. Similarly, the frequency of in-hospital mortality was close to 11% in patients with six complexity factors (chi-squared for trend P < .001) (Fig 1).

### Risk factors associated with AEs and in-hospital mortality

The results of the multivariate logistic regression analysis for all CCIF and other clinical characteristics potentially associated with AEs and in-hospital mortality are summarized in Table 3. Factors from all five domains were associated with AEs and in-hospital mortality.

**Table 3:** Multivariate analysis of care complexity individual factors of adult hospitalized patients associated with adverse events and in-hospital mortality.

Care complexity individual factors	Adverse event^a^			Pressure ulcer^b^			Falls^c^			Aspiration pneumonia^d^			In-hospital mortality^e^		
	n = 3,973 (2.2)			n = 1,673 (0.9)			n = 1,217(0.7)			n = 1,236 (0.7)			n = 3,996 (2.2)		
	OR	(95% CI)	*p-value*	OR	(95% CI)	*p-value*	OR	(95% CI)	*p-value*	OR	(95% CI)	*p-value*	OR	(95% CI)	*p-value*
**Mental-cognitive**															
Agitation	1.14	(0.93–1.38)	0.20	0.91	(0.67–1.25)	0.57	1.36	(0.98–1.91)	0.07	1.20	(0.91–1.58)	0.20	1.10	(0.90–1.34)	0.35
Mental status impairments	2.56	(2.33–2.81)	<0.001	2.30	(2.00–2.65)	<0.001	1.68	(1.40–2.02)	<0.001	3.79	(3.24–4.43)	<0.001	3.83	(3.50–4.18)	<0.001
Impaired cognitive functions	1.53	(0.98–2.39)	0.06	-	-	-	-	-	-	2.02	(1.14–3.60)	0.02	-	-	-
Perception of reality disorders	0.79	(0.53–1.17)	0.23	1.71	(1.06–2.74)	0.02	-	-	-	-	-	-	0.85	(0.57–1.28)	0.45
**Psycho-emotional**															
Aggressive behavior	0.88	(0.61–1.26)	0.47	1.09	(0.65–1.83)	0.75	-	-	-	0.83	(0.49–1.41)	0.49	0.36	(0.21–0.59)	<0.001
Fear/anxiety	1.04	(0.94–1.15)	0.49	0.86	(0.73–1.01)	0.07	1.26	(1.06–1.50)	0.01	0.97	(0.82–1.15)	0.75	0.79	(0.70–0.88)	<0.001
Impaired adaptation	1.36	(1.23–1.52)	<0.001	1.17	(1.00–1.38)	0.05	1.40	(1.15–1.70)	0.001	1.53	(1.31–1.80)	<0.001	1.21	(1.09–1.35)	<0.001
**Sociocultural**															
Language barriers	0.70	(0.38–1.30)	0.26	-	-	-	-	-	-	-	-	-	-	-	-
Social exclusion	1.46	(0.72–2.97)	0.29	-	-	-	-	-	-	-	-	-	-	-	-
Belief conflict	1.21	(0.63–2.33)	0.57	-	-	-	-	-	-	-	-	-	1.36	(0.69–2.68)	0.37
Lack of caregiver support	1.40	(1.24–1.58)	<0.001	1.39	(1.16–1.66)	<0.001	1.58	(1.28–1.96)	<0.001	1.26	(1.04–1.51)	0.02	1.34	(1.19–1.51)	<0.001
**Developmental**															
Old age (≥75 years)	1.91	(1.78–2.06)	<0.001	2.36	(2.11–2.64)	<0.001	1.69	(1.49–1.91)	<0.001	1.54	(1.36–1.75)	<0.001	2.37	(2.20–2.55)	<0.001
Adolescence (17–19 years)	-	-	-	-	-	-	-	-	-	-	-	-	-	-	-
**Comorbidity/complications**															
Major chronic disease	1.15	(1.07–1.23)	<0.001	1.23	(1.11–1.37)	<0.001	1.28	(1.14–1.44)	<0.001	0.99	(0.88–1.11)	0.87	2.27	(2.12–2.44)	<0.001
Hemodynamic instability	1.75	(1.61–1.90)	<0.001	1.76	(1.54–2.00)	<0.001	2.15	(1.85–2.51)	<0.001	1.69	(1.46–1.95)	<0.001	1.36	(1.26–1.46)	<0.001
High-risk of hemorrhage	1.03	(0.84–1.26)	0.77	0.90	(0.66–1.24)	0.52	1.34	(0.99–1.81)	0.06	0.88	(0.62–1.27)	0.51	1.01	(0.84–1.22)	0.91
Communication disorders	1.88	(1.70–2.08)	<0.001	1.36	(1.16–1.60)	<0.001	1.14	(0.92–1.40)	0.22	3.11	(2.68–3.60)	<0.001	1.80	(1.62–2.00)	<0.001
Urinary or fecal incontinence	1.60	(1.46–1.75)	<0.001	1.95	(1.71–2.22)	<0.001	1.11	(0.93–1.33)	0.25	1.48	(1.28–1.72)	<0.001	1.42	(1.30–1.55)	<0.001
Vascular fragility	1.22	(1.06–1.42)	0.007	1.30	(1.07–1.59)	0.01	1.12	(0.85–1.48)	0.43	1.07	(0.83–1.37)	0.61	1.85	(1.64–2.10)	<0.001
Position impairment	1.30	(1.14–1.48)	<0.001	1.57	(1.32–1.87)	<0.001	1.16	(0.90–1.50)	0.25	1.12	(0.90–1.39)	0.32	1.06	(0.92–1.22)	0.41
Involuntary movements	1.25	(0.93–1.69)	0.13	1.01	(0.63–1.62)	0.96	1.01	(0.59–1.90)	0.86	1.51	(1.01–2.27)	0.04	0.94	(0.67–1.31)	0.70
Extreme weight	1.18	(1.04–1.34)	0.009	1.69	(1.43–2.00)	<0.001	0.73	(0.56–0.96)	0.02	1.02	(0.82–1.27)	0.83	1.17	(1.04–1.33)	0.01
Dehydration	-	-	-	-	-	-	-	-	-	-	-	-	2.25	(1.26–4.02)	0.006
Edema	1.13	(0.89–1.43)	0.32	1.40	(1.03–1.90)	0.03	1.01	(0.66–1.58)	0.95	0.89	(0.56–1.41)	0.62	1.73	(1.44–2.01)	<0.001
Uncontrolled pain	1.40	(1.31–1.51)	<0.001	1.61	(1.44–1.79)	<0.001	1.74	(1.54–1.96)	<0.001	1.07	(0.94–1.22)	0.32	1.62	(1.51–1.74)	<0.001
Transmissible infection	1.53	(1.36–1.72)	<0.001	2.33	(2.00–2.70)	<0.001	1.21	(0.97–1.51)	0.09	1.00	(0.79–1.27)	0.97	1.01	(0.88–1.16)	0.84
Immunosuppression	1.33	(0.90–1.98)	0.15	-	-	-	2.17	(1.30–3.68)	0.004	-	-	-	1.30	(0.85–2.00)	0.23
Anatomical and functional disorders	1.23	(1.12–1.35)	<0.001	1.39	(1.22–1.60)	<0.001	1.25	(1.06–1.48)	0.009	0.93	(0.78–1.10)	0.38	0.88	(0.80–0.98)	0.01
Male sex	1.23	(1.15–1.32)	<0.001	1.14	(1.02–1.26)	0.02	1.32	(1.17–1.49)	<0.001	1.34	(1.19–1.51)	<0.001	1.24	(1.16–1.32)	<0.001
High-tech hospital	1.28	(1.19–1.37)	<0.001	1.10	(0.99–1.22)	0.09	1.36	(1.20–1.53)	<0.001	1.47	(1.30–1.66)	<0.001	0.89	(0.83–0.95)	<0.001
Medical ward	1.72	(1.59–1.86)	<0.001	1.36	(1.21–1.53)	<0.001	1.65	(1.45–1.88)	<0.001	2.24	(1.94–2.59)	<0.001	2.33	(2.15–2.54)	<0.001
Length of stay	1.04	(1.04–1.05)	<0.001	1.04	(1.04–1.04)	<0.001	1.02	(1.02–1.03)	<0.001	1.02	(1.02–1.02)	<0.001	1.02	(1.01–1.02)	<0.001

Abbreviations: OR, odds ratio; CI, confidence interval.

^a^ The area under ROC curve of the AEs model was 0.82 (95% IC 0.82–0.83). Multivariate analysis included: all the 28-CCIF (except adolescence), and possible confounders (male sex, high-tech hospital, medical ward and length of stay).

^b^ The area under ROC curve of the pressure ulcer model was 0.88 (95% IC 0.87–0.89). Multivariate analysis included: all the 28-CCIF (except: impaired cognitive functions, language barriers, social exclusion, belief conflict, adolescence, dehydration and immunosuppression), and possible confounders (male sex, high-tech hospital, medical ward and length of stay).

^c^ The area under ROC curve of the falls model was 0.76 (95% IC 0.74–0.77). Multivariate analysis included: all the 28-CCIF (except: impaired cognitive functions, perception of reality disorders, aggressive behavior, language barriers, social exclusion, belief conflict, adolescence and dehydration), and possible confounders (male sex, high-tech hospital, medical ward and length of stay).

^d^ The area under ROC curve of the aspiration pneumonia model was 0.77 (95% IC 0.75–0.78). Multivariate analysis included: all the 28-CCIF (except: perception of reality disorders, language barriers, social exclusion, belief conflict, adolescence, dehydration and immunosuppression), and possible confounders (male sex, high-tech hospital, medical ward and length of stay).

^e^ The area under ROC curve of the in-hospital mortality model was 0.79 (95% IC 0.78–0.79). Multivariate analysis included: all the 28-CCIF (except: impaired cognitive functions, language barriers, social exclusion and adolescence), and possible confounders (male sex, high-tech hospital, medical ward and length of stay).

After adjustment for potential confounders (gender, length of stay, hospital level, and medical ward), multivariate logistic regression analysis of AEs showed independent associations with impaired mental status, impaired adaptation, lack of caregiver support, old age, major chronic disease, hemodynamic instability, communication disorders, urinary or fecal incontinence, vascular fragility, position impairment, extreme weight, uncontrolled pain, transmissible infection, anatomical and functional disorders, male sex, length of hospital stay, admission to a medical ward and to a high-tech hospital. The area under the ROC curve was 0.82 (95% confidence interval [CI]: 0.82–0.83).

In the multivariate logistic regression of individual AEs, pressure ulcer obtained similar results, including perception of reality disorders and edema as risk factors. The area under the ROC curve was 0.88 (95% CI: 0.87–0.89).

Regarding falls, fear/anxiety and immunosuppression were additional independent factors (area under the ROC curve 0.76 [95% CI: 0.74–0.77]).

Both involuntary movements and impaired cognitive function were independent risk factors associated with aspiration pneumonia (area under the ROC curve 0.77 [95% CI: 0.75–0.78]).

Finally, a multivariate logistic regression of factors associated with in-hospital mortality was performed. In addition to factors previously associated with AEs, dehydration and edema were risk factors independently associated with in-hospital mortality. Moreover, aggressive behavior, fear/anxiety, anatomical and functional disorders and high-tech hospital admission were protective factors associated with in-hospital mortality. The area under the ROC curve was 0.79 (95% CI: 0.78–0.79). The AUC of the five outcomes analyzed were > 0.75, showing a fair discriminatory power.

## Discussion

In this study of a large number of hospitalized patients, we found that a considerable proportion presented one of the AEs studied (pressure ulcer, falls and aspiration pneumonia) or died during hospitalization. The risk factors independently associated with both AE and in-hospital mortality were mental status impairments, impaired adaptation, lack of caregiver support, old age, major chronic disease, hemodynamic instability, communication disorders, urinary or fecal incontinence, vascular fragility, extreme weight, uncontrolled pain, male sex, length of stay and admission to a medical ward. High-tech hospital admission was associated with an increased risk of adverse events and a reduced risk of in-hospital mortality.

Around 2% of patients experienced an AE, a frequency similar to those recorded in previous reports [7,11,26–29]. In addition, 2.2% of patients died during hospitalization, again in line with other studies reporting rates ranging between 1–5% [9,30,31]. Nevertheless, those studies rated the selected AEs in the context of medical conditions and did not account for psychosocial factors.

To the best of our knowledge, this is the first study of CCIF that has included broader health, functional, and psychosocial problems in order to identify the risk factors associated with selected AEs and in-hospital mortality. After adjustment for potential confounders in the multivariate analysis, we found that several CCIF from all domains were associated with AEs and in-hospital mortality.

Mental status impairments, impaired adaptation, lack of caregiver support, old age, major chronic disease, hemodynamic instability, communication disorders, urinary or fecal incontinence, vascular fragility, position impairment, extreme weight, uncontrolled pain, transmissible infection, anatomical and functional disorders, male sex, length of hospital stay, admission to a medical ward and, high-tech hospital were independent factors associated with AEs.

Regarding age, older patients exhibit a higher risk of becoming frail, of presenting comorbidities and experiencing AEs during hospitalization, probably because of their increased care needs [11,26,32,33]. A systematic review identified co-morbidity, reduced functional ability and lower quality of care as important causative factors associated with AEs [11]. The results of that study coincide with our in identifying old age and major chronic disease as risk factors associated with AEs. Regarding functional ability, our study also identified position impairment and anatomical-functional disorders as independent factors associated with AEs.

Furthermore, previous studies have stressed that frequent patient surveillance and measurement of vital signs are crucial for the early detection of acute deterioration [34], and have identified an association between hemodynamic instability and poor outcomes [19,35]. Therefore, patients with hemodynamic instability or uncontrolled pain [36] are probably at a high risk of suffering AEs during hospitalization and require a longer hospital stay.

Similarly, isolated patients are at a higher risk of AEs such as hospital-acquired pressure ulcers [37]. Our findings are also consistent with previous studies showing that mental status impairments are associated with hospital-acquired complications [38,39]. Furthermore, other studies have shown that frail people admitted to hospitals can develop geriatric syndromes, with a higher occurrence of CCIF such as communication disorders, incontinence, vascular fragility or nutritional syndromes [4,11,40–43].

Impaired adaptation, disruptive behaviors, hopelessness or powerlessness may also affect patients’ recovery, generating feelings of exclusion, self-blame, frustration or loss of control, and negatively impacting overall health outcomes [19,44,45]. Our study also found that lack of caregiver support during hospitalization was associated with AEs. In this regard, unpaid caregivers such as family members or friends often take on an active caregiver role in hospital to mitigate the risk of functional decline, falls, and hospital-related adverse events [46]. Previous studies have shown that psychoeducational interventions aimed at caregivers reduce burden and emotional distress and enhanced caregivers' perceived social support [47]. Future studies should assess the impact of psychosocial interventions and advanced nursing care on patients’ and caregivers’ health outcomes.

Mental-cognitive factors such as perception of reality disorders and impaired cognition were independently associated with pressure ulcers and aspiration pneumonia. These results corroborate those of previous inquiries that identified delirium as a risk factor for these AEs [33,40]. Within the psycho-emotional domain, fear and anxiety were risk factors for falls. Previous studies have shown that patients with fear of falling had a higher average of falls [48]. Conversely, our study identified fear/anxiety and aggressive behavior as protective factors against in-hospital mortality. Patients with severe illness often experienced anxiety due to their vulnerable situation [49]. Our univariate analysis showed a higher frequency of fear/anxiety in patients who finally died compared with patients discharged alive; however, these results should be corroborated with an analysis of long-term mortality. Similarly, aggressive behavior was more frequent among patients admitted to psychiatric units, and lower frequency of in-hospital mortality was observed in these wards. Furthermore, in the general population and also among in-patients, aggressiveness may be considered a “behavioral response of incitement and attack, verbal and/or physical, due to a stressful situation, loss of control emotional disorganization or intolerance to frustration; it is an acute situational, reactive adaption response or coping strategy” [24]. Aggressiveness is related to violence but is not synonymous with it; in violence there exists intentionality to harm oneself or others, while aggressive behavior may merely be a coping strategy in a particular situation. It has certain positive qualities such as helping to focus on one’s own values and goals to adjust to new realities and refusing to simply accept things in a passive way. In this sense, aggressiveness, while not a positive behavior in itself, might have a protective effect [24].

Other comorbidity-complication factors were independently associated with the AEs selected: edema with pressure ulcers, immunosuppression with falls, and involuntary movements with aspiration pneumonia. First, edema is a strong risk factor for hospitalization [50] and skin tear development [51]. Second, immunosuppressed patients have an increased risk of falls due to disease or treatment-related consequences such as muscle weakness, joint impairment, reduced mobility and postural instability [52]. Third, involuntary movements and muscle weakness are common in patients with neurodegenerative diseases, increasing the risk of aspiration due to swallowing dysfunction [53]. Additionally, dehydration was a risk factor for in-hospital mortality while anatomical and functional disorders were protective factors. Some studies have shown that dehydration is closely linked to an increased risk of mortality [54], while anatomical and functional disorders include specifications such as amputations, deformities and joint stiffness which are associated with admission profiles with low risk of severity and mortality (APR-GRD 1–2).

Finally, we found that length of stay, male sex and admission to the medical ward were independent factors associated with AEs and in-hospital mortality. Previous studies have found that length of stay and comorbidities were higher among patients who died during hospitalization [9] or who experienced AEs [7]. Moreover, other studies show that mortality was higher among male, mainly due to cardiovascular diseases [55]. Similarly, patients admitted to medical wards tended to have poor health outcomes [19].

Finally, admission to a high-tech hospital was a risk factor associated with AEs and a protective factor for in-hospital mortality. Several studies have demonstrated the association between acute status and other patient and organizational measures, and nurse-sensitive outcomes such as mortality and the AE studied here. Nevertheless, further studies are required that combine acuity and complexity measures, as well as organizational variables such as nurse staffing or missed care, to gain a better understanding of their role in patient outcomes [56–58].

The strengths of this study are its case-control design and the large number of patients included. Moreover, CCIF and clinical data were comprehensively collected from electronic health record systems and the data warehouse of the Catalan Institute of Health. Physiological, mental-cognitive and sociocultural factors were included in order to identify broader health contributors to AEs and in-hospital mortality. However, there are also some limitations that should be acknowledged. Data on all variables included in the nursing assessments in the electronic health records are usually collected at the time of patient admission in the ward and must be re-evaluated during hospitalization depending on the patient’s needs. Therefore, we assumed proper compliance with electronic health records and administrative data; however, voluntary completion of electronic health records probably provides close-to-reality information on nurses’ observations and judgements on patient status and progress, but not the reality in itself [59]. In addition, this is not a matched case-control study and we did not evaluate other confounders such as patient’s lifestyle or Charlson comorbidity index. Finally, we did not consider other AEs known to be sensitive to nursing care in other studies, such as risk of caregiver compassion fatigue [58].

Our study highlights several points that should be taken into account during patient care and may help to identify factors associated with nurse-sensitive patient outcomes, as described in previous studies [4]. Based on these results, nurses should identify patient complexity factors during admission, and those that are modifiable such as hemodynamic instability on an ongoing basis, in order to implement an effective care process and to prevent poor outcomes. CCIF related to psycho-emotional needs and sociocultural factors may play an important role in healthcare outcomes. Overall, our study found that the frequency of AEs and in-hospital mortality rose with increasing numbers of risk factors and surpassed 10% in patients with at least six CCIF. Our findings may contribute to identifying and stratifying the risk of patient in-hospital outcomes. Nurses have a key role in assessing CCIF and in conducting prevention-oriented interventions, including frequent patient surveillance to minimize potential AEs and mortality.

## Conclusions

The risk factors independently associated with both AE and in-hospital mortality were mental status impairments, impaired adaptation, lack of caregiver support, old age, major chronic disease, hemodynamic instability, communication disorders, urinary or fecal incontinence, vascular fragility, extreme weight, uncontrolled pain, male sex, length of stay and admission to medical ward. High-tech hospital admission was associated with an increased risk of adverse events and a reduced risk of in-hospital mortality.

## References

[1] King A, Bottle A, Faiz O, Aylin P. Investigating Adverse Event Free Admissions in Medicare Inpatients as a Patient Safety Indicator. Ann Surg. 2017;265: 910–915. 10.1097/SLA.0000000000001792 27192350

[2] Smith M, Saunders R, Stuckhardt L, McGinnis J. Best Care at Lower Cost The Path to Continuously Learning Health Care in America Committee on the Learning Health Care System in America. Washington (DC): National Academies Press (US); 2013 10.17226/13444 24901184

[3] Silva L, Terra F, Macedo F, Santos S, Batista M. Notification of adverse events: characterization of events occurred in a hospital institution. J Nurs UFPE. 2014;8: 3015–23. 10.5205/1981-8963-v8i9a10020p3015-3023-2014

[4] Bail K, Grealish L. ‘Failure to Maintain’: A theoretical proposition for a new quality indicator of nurse care rationing for complex older people in hospital. Int J Nurs Stud. 2016;63: 146–161. 10.1016/j.ijnurstu.2016.08.001 27658271

[5] Hauck K, Zhao X, Jackson T. Adverse event rates as measures of hospital performance. Health Policy (New York). 2012;104: 146–154. 10.1016/j.healthpol.2011.06.010 21782269

[6] Wang X, Liu K, You LM, Xiang JG, Hu HG, Zhang LF, et al The relationship between patient safety culture and adverse events: A questionnaire survey. Int J Nurs Stud. 2014;51: 1114–1122. 10.1016/j.ijnurstu.2013.12.007 24418106

[7] Rafter N, Hickey A, Condell S, Conroy R, O’connor P, Vaughan D, et al Adverse events in healthcare: Learning from mistakes. Q J Med. 2015;108: 273–277. 10.1093/qjmed/hcu145 25078411

[8] Duarte S da CM, Stipp MAC, da Silva MM, de Oliveira FT. Adverse events and safety in nursing care. Rev Bras Enferm. 2015;68: 136–146. 10.1590/0034-7167.2015680120p 25946507

[9] Flaatten H, Brattebø G, Alme B, Berge K, Rosland JH, Viste A, et al Adverse events and in-hospital mortality: An analysis of all deaths in a Norwegian health trust during 2011. BMC Health Serv Res. 2017;17: 1–7. 10.1186/s12913-016-1943-z 28683802 PMC5501336

[10] Zegers M, De Bruijne MC, Wagner C, Hoonhout LHF, Waaijman R, Smits M, et al Adverse events and potentially preventable deaths in Dutch hospitals: Results of a retrospective patient record review study. Qual Saf Heal Care. 2009;18: 297–302. 10.1136/qshc.2007.025924 19651935

[11] Long SJ, Brown KF, Ames D, Vincent C. What is known about adverse events in older medical hospital inpatients? A systematic review of the literature. Int J Qual Heal Care. 2013;25: 542–554. 10.1093/intqhc/mzt056 23925507

[12] Hoogerduijn JG, Grobbee DE, Schuurmans MJ. Prevention of functional decline in older hospitalized patients: Nurses should play a key role in safe and adequate care. Int J Nurs Pract. 2014;20: 106–113. 10.1111/ijn.12134 24580981

[13] Parke B, Hunter KF, Strain LA, Marck PB, Waugh EH, McClelland AJ. Facilitators and barriers to safe emergency department transitions for community dwelling older people with dementia and their caregivers: A social ecological study. Int J Nurs Stud. 2013;50: 1206–1218. 10.1016/j.ijnurstu.2012.11.005 23219329

[14] Roberson D, Connell M, Dillis S, Gauvreau K, Gore R, Heagerty E, et al Cognitive complexity of the medical record is a risk factor for major adverse events. Perm J. 2014;18: 4–8. 10.7812/tpp/12-142 24626065 PMC3951023

[15] Hong CS, Atlas SJ, Ashburner JM, Chang Y, He W, Ferris TG, et al Evaluating a Model to Predict Primary Care Physician-Defined Complexity in a Large Academic Primary Care Practice-Based Research Network. J Gen Intern Med. 2015;30: 1741–1747. 10.1007/s11606-015-3357-8 26048275 PMC4636571

[16] Zullig LL, Whitson HE, Hastings SN, Beadles C, Kravchenko J, Akushevich I, et al A Systematic Review of Conceptual Frameworks of Medical Complexity and New Model Development. J Gen Intern Med. 2016;31: 329–337. 10.1007/s11606-015-3512-2 26423992 PMC4762821

[17] Safford MM. The Complexity of Complex Patients. J Gen Intern Med. 2015;30: 1724–1725. 10.1007/s11606-015-3472-6 26259761 PMC4636570

[18] Juvé-Udina M.E., Matud C., Farrero S., Jiménez H., Rodríguez E., Martínez M. et al Intensity of nursing care: workloads or individual complexity? Metas de Enfermería. 2010;13: 6–14.

[19] Adamuz J, González-Samartino M, Jiménez-Martínez E, Tapia-Pérez M, López-Jiménez MM, Ruiz-Martínez MJ, et al Care Complexity Individual Factors Associated With Hospital Readmission: A Retrospective Cohort Study. J Nurs Scholarsh. 2018;50: 411–421. 10.1111/jnu.12393 29920928

[20] Souza J, Santos JV, Canedo VB, Betanzos A, Alves D, Freitas A. Importance of coding co-morbidities for APR-DRG assignment: Focus on cardiovascular and respiratory diseases. Heal Inf Manag J. 2020;49: 47–57. 10.1177/1833358319840575 31043088

[21] Montes-Santiago J, Rodil V, Formiga F, Cepeda JM, Urrutia A. Características y costes de los pacientes ingresados por arritmias cardiacas en España. Rev Clin Esp. 2013;213: 235–239. 10.1016/j.rce.2013.02.003 23561445

[22] Ministerio de Sanidad SS e I. Ministerio de Sanidad, Consumo y Bienestar Social—Estadísticas / Estudios—Sistema de Información Sanitaria del SNS In: Registro de altas. ICMBD: Indicadores y ejes de análisis del CMBD. Madrid: Aplicación para el análisis y explotación del registro de altas hospitalarias [Internet]. 2015 [cited 6 Feb 2020]. Available: http://icmbd.es/login-success.do

[23] Aiken LH, Sloane D, Griffiths P, Rafferty AM, Bruyneel L, McHugh M, et al Nursing skill mix in European hospitals: Cross-sectional study of the association with mortality, patient ratings, and quality of care. BMJ Qual Saf. 2017;26: 559–568. 10.1136/bmjqs-2016-005567 28626086 PMC5477662

[24] Juvé-Udina ME. What patients’ problems do nurses e-chart? Longitudinal study to evaluate the usability of an interface terminology. Int J Nurs Stud. 2013;50: 1698–1710. 10.1016/j.ijnurstu.2013.04.008 23684394

[25] Vittinghoff E, McCulloch CE. Relaxing the rule of ten events per variable in logistic and cox regression. Am J Epidemiol. 2007;165: 710–718. 10.1093/aje/kwk052 17182981

[26] Thomas EJ, Brennan TA. Incidence and types of preventable adverse events in elderly patients: Population based review of medical records. BMJ. 2000;162: 2725 10.1136/bmj.320.7237.741 10720355 PMC27315

[27] González-Samartino M, Delgado-Hito P, Adamuz-Tomás J, Cano MFV, Creus MC, Juvé-Udina ME. Accuracy and completeness of records of adverse events through interface terminology. Rev da Esc Enferm. 2018;52: e03306 10.1590/S1980-220X2017011203306 29668785

[28] Kirshenbaum EJ, Blackwell RH, Li B, Kothari AN, Kuo PC, Flanigan RC, et al Implications of postoperative pulmonary aspiration following major urologic surgery. Can J Urol. 2018;25: 9186–9192. 29524973

[29] Schiek S, Hildebrandt K, Zube O, Bertsche T. Fall-risk-increasing adverse reactions—is there value in easily accessible drug information? A case-control study. Eur J Clin Pharmacol. 2019;75: 849–857. 10.1007/s00228-019-02628-x 30758518

[30] Moore BJ, White S, Washington R, Coenen N, Elixhauser A. Identifying Increased Risk of Readmission and In-hospital Mortality Using Hospital Administrative Data: The AHRQ Elixhauser Comorbidity Index. Med Care. 2017;55: 698–705. 10.1097/MLR.0000000000000735 28498196

[31] Conway R, Byrne D, O’Riordan D, Silke B. Outcomes in acute medicine—Evidence from extended observations on readmissions, hospital length of stay and mortality outcomes. Eur J Intern Med. 2019;66: 69–74. 10.1016/j.ejim.2019.06.001 31196741

[32] Albolino S, Tartaglia R, Bellandi T, Bianchini E, Fabbro G, Forni S, et al Variability of adverse events in the public health-care service of the Tuscany region. Intern Emerg Med. 2017;12: 1033–1042. 10.1007/s11739-017-1698-5 28646442

[33] Byun SE, Shon HC, Kim JW, Kim HK, Sim Y. Risk factors and prognostic implications of aspiration pneumonia in older hip fracture patients: A multicenter retrospective analysis. Geriatr Gerontol Int. 2019;19: 119–123. 10.1111/ggi.13559 30556343

[34] Stevenson JE, Israelsson J, Nilsson GC, Petersson GI, Bath PA. Recording signs of deterioration in acute patients: The documentation of vital signs within electronic health records in patients who suffered in-hospital cardiac arrest. Health Informatics J. 2016;22: 21–33. 10.1177/1460458214530136 24782478

[35] Juvé-Udina ME, Fabrellas-Padrés N, Adamuz-Tomás J, Cadenas-González S, Gonzalez-Samartino M, Ariza L de la C, et al Surveillance nursing diagnoses, ongoing assessment and outcomes on in-patients who suffered a cardiorespiratory arrest. Rev da Esc Enferm. 2017;51: e03286 10.1590/S1980-220X2017004703286 29562038

[36] Jeon N, Sorokina M, Henriksen C, Staley B, Lipori GP, Winterstein AG. Measurement of selected preventable adverse drug events in electronic health records: Toward developing a complexity score. Am J Heal Pharm. 2017;74: 1865–1877. 10.2146/ajhp160911 29118045

[37] Hyun S, Moffatt-Bruce S, Cooper C, Hixon B, Kaewprag P. Prediction model for hospital-acquired pressure ulcer development: New paradigm in intensive care units. J Med Internet Res. 2019;21: e13785 10.2196/13785 31322127 PMC6670273

[38] Butcher L. Caring for patients with dementia in the acute care setting. Br J Nurs. 2018;27: 358–362. 10.12968/bjon.2018.27.7.358 29634328

[39] Bail K, Draper B, Berry H, Karmel R, Goss J. Predicting excess cost for older inpatients with clinical complexity: A retrospective cohort study examining cognition, comorbidities and complications. PLoS One. 2018;13: e0193319 10.1371/journal.pone.0193319 29474407 PMC5825075

[40] Lannering C, Ernsth Bravell M, Midlöv P, Östgren CJ, Mölstad S. Factors related to falls, weight-loss and pressure ulcers—more insight in risk assessment among nursing home residents. J Clin Nurs. 2016;25: 940–950. 10.1111/jocn.13154 26813994

[41] Hubbard RE, Peel NM, Samanta M, Gray LC, Mitnitski A, Rockwood K. Frailty status at admission to hospital predicts multiple adverse outcomes. Age Ageing. 2017;46: 801–806. 10.1093/ageing/afx081 28531254

[42] Bell SP, Vasilevskis EE, Saraf AA, Jacobsen JML, Kripalani S, Mixon AS, et al Geriatric Syndromes in Hospitalized Older Adults Discharged to Skilled Nursing Facilities. J Am Geriatr Soc. 2016;64: 715–722. 10.1111/jgs.14035 27059831 PMC4840035

[43] Farage MA, Miller KW, Berardesca E, Maibach HI. Clinical implications of aging skin: Cutaneous disorders in the elderly. Am J Clin Dermatol. 2009;10: 73–86. 10.2165/00128071-200910020-00001 19222248

[44] Finke EH, Light J, Kitko L. A systematic review of the effectiveness of nurse communication with patients with complex communication needs with a focus on the use of augmentative and alternative communication. J Clin Nurs. 2008;17: 2102–2115. 10.1111/j.1365-2702.2008.02373.x 18705734

[45] Juvé-Udina ME, Pérez EZ, Padrés NF, Samartino MG, García MR, Creus MC, et al Basic nursing care: Retrospective evaluation of communication and psychosocial interventions documented by nurses in the acute care setting. J Nurs Scholarsh. 2014;46: 65–72. 10.1111/jnu.12062 24354414

[46] Everall AC, Guilcher SJT, Cadel L, Asif M, Li J, Kuluski K. Patient and caregiver experience with delayed discharge from a hospital setting: A scoping review. Heal Expect. 2019;22: 863–873. 10.1111/hex.12916 31099969 PMC6803563

[47] Zabalegui A, Galisteo M, Navarro MM, Cabrera E. INFOSA intervention for caregivers of the elderly, an experimental study. Geriatr Nurs (Minneap). 2016;37: 426–433. 10.1016/j.gerinurse.2016.06.001 27477085

[48] Visschedijk JHM, Caljouw MAA, Bakkers E, Van Balen R, Achterberg WP. Longitudinal follow-up study on fear of falling during and after rehabilitation in skilled nursing facilities Physical functioning, physical health and activity. BMC Geriatr. 2015;15: 161 10.1186/s12877-015-0158-1 26637334 PMC4670507

[49] Strang S, Ekberg-Jansson A, Henoch I. Experience of anxiety among patients with severe COPD: A qualitative, in-depth interview study. Palliat Support Care. 2013;12: 465–472. 10.1017/S1478951513000369 23916195 PMC4413871

[50] Voors AA, Ouwerkerk W, Zannad F, van Veldhuisen DJ, Samani NJ, Ponikowski P, et al Development and validation of multivariable models to predict mortality and hospitalization in patients with heart failure. Eur J Heart Fail. 2017;19: 627–634. 10.1002/ejhf.785 28247565

[51] Lewin GF, Newall N, Alan JJ, Carville KJ, Santamaria NM, Roberts PA. Identification of risk factors associated with the development of skin tears in hospitalised older persons: a case-control study. Int Wound J. 2016;13: 1246–1251. 10.1111/iwj.12490 26400842 PMC7949919

[52] Lourenço M de A, Carli FVBO, de Assis MR. Characterization of falls in adults with established rheumatoid arthritis and associated factors. Adv Rheumatol. 2018;58: 16 10.1186/s42358-018-0021-0 30657096

[53] Umemoto G, Furuya H. Management of dysphagia in patients with Parkinson’s disease and related disorders. Intern Med. 2020;59: 7–14. 10.2169/internalmedicine.2373-18 30996170 PMC6995701

[54] Sheills R, Morrell-Scott N. Prevention of dehydration in hospital patients. Br J Nurs. 2018;27: 565–569. 10.12968/bjon.2018.27.10.565 29791217

[55] Maiolo V, Reid AM. Looking for an explanation for the excessive male mortality in England and Wales since the end of the 19th century. SSM—Popul Heal. 2020;11: 100584 10.1016/j.ssmph.2020.100584 32346599 PMC7178544

[56] Juvé-Udina M-E, Adamuz J, López-Jimenez M-M, Tapia-Pérez M, Fabrellas N, Matud-Calvo C, et al Predicting patient acuity according to their main problem. J Nurs Manag. 2019;27: 1845–1858. 10.1111/jonm.12885 31584733 PMC7328732

[57] Lasater KB, McHugh M, Rosenbaum PR, Aiken LH, Smith H, Reiter JG, et al Valuing hospital investments in nursing: Multistate matched-cohort study of surgical patients. BMJ Qual Saf. 2020 10.1136/bmjqs-2019-010534 32220938 PMC7530638

[58] Juvé-Udina M, González-Samartino M, López-Jiménez MM, Planas-Canals M, Rodríguez-Fernández H, Batuecas-Duelt IJ, et al Acuity, nurse staffing and workforce, missed care and patient outcomes. A cluster-unit-level descriptive comparison. J Nurs Manag. 2020 [cited 11 May 2020]. 10.1111/jonm.13040 32384199 PMC7754324

[59] Juvé-Udina ME, Pérez EZ, Padrés NF, Samartino MG, García MR, Creus MC, et al Basic nursing care: Retrospective evaluation of communication and psychosocial interventions documented by nurses in the acute care setting. J Nurs Scholarsh. 2014;46: 65–72. 10.1111/jnu.12062 24354414

